# No Evidence of the Effect of the Interventions to Combat Health Care Fraud and Abuse: A Systematic Review of Literature

**DOI:** 10.1371/journal.pone.0041988

**Published:** 2012-08-24

**Authors:** Arash Rashidian, Hossein Joudaki, Taryn Vian

**Affiliations:** 1 Department of Health Management and Economics, School of Public Health, Tehran, Iran; 2 Knowledge Utilization Research Center, Tehran University of Medical Sciences, Tehran, Iran; 3 Department of Health Management and Economics, School of Public Health, Tehran University of Medical Sciences, Tehran, Iran; 4 School of Public Health, Boston University, Boston, Massachusetts, United States of America; Tehran University of Medical Sciences, Iran (Islamic Republic of)

## Abstract

**Background:**

Despite the importance of health care fraud and the political, legislative and administrative attentions paid to it, combating fraud remains a challenge to the health systems. We aimed to identify, categorize and assess the effectiveness of the interventions to combat health care fraud and abuse.

**Methods:**

The interventions to combat health care fraud can be categorized as the interventions for ‘prevention’ and ‘detection’ of fraud, and ‘response’ to fraud. We conducted sensitive search strategies on Embase, CINAHL, and PsycINFO from 1975 to 2008, and Medline from 1975–2010, and on relevant professional and organizational websites. Articles assessing the effectiveness of any intervention to combat health care fraud were eligible for inclusion in our review. We considered including the interventional studies with or without a concurrent control group. Two authors assessed the studies for inclusion, and appraised the quality of the included studies. As a limited number of studies were found, we analyzed the data using narrative synthesis.

**Findings:**

The searches retrieved 2229 titles, of which 221 full-text studies were assessed. We found no studies using an RCT design. Only four original articles (from the US and Taiwan) were included: two studies within the detection category, one in the response category, one under the detection and response categories, and no studies under the prevention category. The findings suggest that data-mining may improve fraud detection, and legal interventions as well as investment in anti-fraud activities may reduce fraud.

**Discussion:**

Our analysis shows a lack of evidence of effect of the interventions to combat health care fraud. Further studies using robust research methodologies are required in all aspects of dealing with health care fraud and abuse, assessing the effectiveness and cost-effectiveness of methods to prevent, detect, and respond to fraud in health care.

## Introduction

Fraud has been defined as an “intentional deception or misrepresentation made by a person or an entity, with the knowledge that the deception could result in some kinds of unauthorized benefits to that person or entity” [1]. Because of complexities of defining fraudulent behavior and detecting fraudulent cases, measuring fraud losses in health care is difficult [2]. Undetected frauds remain a problem; in many individual cases, it may not be possible to determine whether a claim is fraudulent or not. Still, it has been estimated that three to ten per cent of health care spending is lost to health care fraud and abuse, amounting to billions of dollars per year [3],[4].

In general, fraud incorporates the following elements: misrepresentation of a material fact, knowledge of the falsity of the misrepresentation, intent, a victim that can be a person or organization acting on the misrepresentation, and damage to the victim [5]. Fraud boundaries are confused with other concepts such as error, negligence, abuse, and corruption. Abuse is the closest concept to fraud and usually accompanies it. The degree of intent by the individual or entity is often the determining factor in distinguishing between fraud and abuse. The term “abuse” may be used to describe problematic behavior which is not clearly against the law or where certain elements of the fraud definition (such as knowing deception) are missing [6].

Despite the importance of fraud and the varying levels of political, legislative, and administrative attention paid to it, combating health care fraud remains an important challenge to the health systems. Sparrow (1998), in his review of public sector fraud, suggested that several factors explain why combating fraud is difficult: the amount of fraud identified by the detection systems is never the real scale of the problem; the fraud control performance indicators are ambiguous and misleading; the amount of resources available to combat fraud remains limited; combating fraud is a dynamic process and requires continuous change; and too much reliance is placed on traditional enforcement approaches (such as case-note reviews which are costly to conduct and lack reliability) [2].

Health care systems are particularly vulnerable to fraud and corruption [7]. Many factors including the asymmetry of information between providers and patients, inelastic demand for services, the enormous volume of money spent on health care, the presence of third-party and fee-for-service payments, and the public trust in providers, exacerbate the problem within the health care sector [8],[9].

### Classification and typology of health care fraud

Based on who conducts the fraud, we can classify fraud into categories of provider fraud, consumer fraud (patient or insured), and insurer or payer fraud [10]. Provider health care fraud may be committed by individuals (e.g. physicians, dentists) or by provider organizations (e.g. hospitals). Sometimes providers engage in frauds that involve other service providers (e.g. diagnostic services) or pharmaceutical and medical device manufacturers by receiving kickback payments (See Table 1).

**Table 1:** Some examples of fraud and abuse.

Providers fraud	Patients or insured people fraud	Insurer (third party payer) fraud	Abuse
• Phantom billing: Billing for Services not provided. Adding otherwise legitimate claim charges for services never performed (padding the bill) or fabricating claims.	• Doctor shopping: Bouncing from one doctor to another in order to obtain multiple prescriptions for controlled substances.	• Agent or insurer falsifying reimbursements	• Substandard care: incidents or practices those are not consistent with the standard of care
• Up-coding: Charging for a more expensive service such as a visit to a specialist when the patient actually saw a nurse or an intern.	• Identity theft: Obtaining and using another person's health insurance card or identification, by theft, or deception, to obtain health care or other services or to impersonate that individual.	• Agent or insurer falsifying benefit or service statements	• Providing unnecessary care: Including unnecessary tests, surgeries, and other procedures, for the purpose of increasing the reimbursement.
• Misrepresenting services: Performing uncovered services but billing insurance companies for different services that are covered.	• Misuse of insurance card: allowing some unauthorized person to use your ID card to obtain medical services or drugs. Acting in collusion with the insured/member to obtain health care services by assuming the member's identity	•Agent or insurer collecting premiums, then issuing no insurance	• Unnecessary costs to a program caused either directly or indirectly: via unnecessary care, or additional services not warranted for the well-being or satisfaction of the patient.
• Misrepresenting the Diagnosis to Justify Payment	• Patients claim exemption from prescription charges when they are not in fact exempt.		• Failure to document medical records adequately in the payer's view
• Unbundling or “Exploding” Charges: Charging separately for procedures that are actually part of a single procedure	• Patients have falsely stated that they have lost their prescriptions and obtained duplicates.		• Using insurance for a service that fails to meet coverage requirements
• Falsifying Certificates of Medical Necessity, Plans of Treatment, and Medical Records to Justify Payment	• Patients have falsely registered with a number of doctors and obtained prescriptions from each.		• Charging the insurers higher rates than that for non-insured patients (i.e. normal tariffs)
• Billing for professional services rendered by personnel lacking appropriate credentials.			
• Payment or receiving kickbacks (also known as fee-splitting)			
• Self-referral: referring the patients to a clinic, diagnostic service, hospital etc with which the referring physician has a financial relationship.			

Based on where the fraud happens and the type of services offered, we may relate fraud to entities such as hospitals (inpatient care), clinics (outpatient care, dental care), home care organizations, pharmacies etc. Fraud is also viewed from the angle of the typology of the fraudulent behaviors. In the USA, three types of provider frauds are identified by health care fraud laws: submission of false claims, the payment or receipt of kickbacks, and self-referrals [11].

There are certain types of fraud occurring at the organization level which are not specific to the health care systems, namely financial fraud [12]. Financial fraud is often committed by employees of an organization against their own organizations, usually linked with the accountancy procedures within the organization. Also corruption in health care includes other categories of unlawful behavior (e.g. embezzlement, bribery etc) [13]. Our study focuses on health care fraud only and these types of fraud are beyond our study.

### Objectives

In this research we used systematic review methodology and assessed the effectiveness of the interventions used to combat health care fraud, and aimed to identify the gaps in evidence for future studies.

We categorized all the interventions used to combat health care fraud into three categories: prevention, detection, and response [14]. The categorization is helpful for simplification and understanding of the interventions, while there are links of overlaps between the categories.

## Methods

### Study design

We used systematic review methodology to assess the effectiveness of interventions to combat health care fraud.

### Information sources and search strategies

A sensitive systematic search strategy was devised and run on Medline, Embase, CINAHL, and PsycINFO from 1975 to 2008. The search strategies were tested for their sensitivity of capturing known relevant papers, and were modified accordingly. The general format for the final search strategy was ‘fraud related terms’ and ‘research design terms’. The fraud related terms used in the search included: fraud, forgery, misconduct, deception, falsification, abuse, misrepresentation, unbundling, corruption, kickback, fee splitting, quackery, counterfeit, bribing, and upcoding. The terms were used in various formats and combinations to ensure high sensitivity of the search (See Appendix S1 for an example of a full search strategy). We used design filters as developed by the Cochrane Effective Practice and Organisation of Care Group that included experimental and quasi-experimental research designs [15]. We later updated this search by covering 2008–2010 and searching the Medline database. In addition, we also conducted targeted searches for fraud detection and data mining methods to ensure relevant interventional studies were not missed, as well as general searches of health care fraud in the Google and the Google Scholars search engines to find related reports or conference proceedings. We also searched organizational websites including the National Health Care Anti-Fraud Association (http://www.nhcaa.org), Association of Certified Fraud Examiners (http://www.acfe.org), European Health Care Fraud and Corruption Network (http://www.ehfcn.org), Centers for Medicare and Medicaid Services (http://www.cms.gov), the NHS Counter Fraud Service (http://www.nhsbsa.nhs.uk/CounterFraud.aspx), Coalition Against Insurance Fraud (http://www.insurancefraud.org), and the US Department of Health & Human Services Office of Inspector General (http://oig.hhs.gov). We conducted backward citation search for all included studies and relevant review articles [10],[16].

### Inclusion criteria

Articles that involved an effectiveness study of any intervention to combat health care fraud (including prevention, detection, and response interventions, as defined below) were eligible for inclusion in our study. We considered including the interventional studies with or without a concurrent control group (e.g. uncontrolled before-after studies were eligible for inclusion).

### Classification of the interventions to combat health care fraud

#### a. Interventions aimed at preventing health care fraud

In general, prevention interventions refer to the interventions that deter potential fraudsters from attempting fraud, and stopping a fraud attempt before the fraud is actually committed. Creating an anti-fraud culture (i.e. changing the beliefs, attitudes, social norms, and cultural factors; e.g. changing perceptions such as “fraud will not be detected or punished” and “insurance fraud is a victimless crime”) [17], and improving internal control and developing ‘compliance systems’ (e.g. hospital compliance programs in the USA) [18],[19] are two general approaches used in designing the interventions to prevent health care fraud. ‘Compliance systems’ are defined as “systematic processes aimed at ensuring that the organization and its employees (and perhaps business partners) comply with applicable laws, regulations, and standards. In the context of health care, it usually includes a comprehensive strategy to ensure the submission of consistently accurate claims to federal, state, and commercial payers.” [18].

#### b. Interventions aimed at detecting health care fraud

Fraud detection involves identifying past and new cases of fraud as quickly as possible after a fraud has been committed. It includes (manual) retrospective medical claims' reviews [20]–[22]. Computer systems for reviewing medical claims that work based on predefined rules and algorithms new methods of health care fraud detection based on artificial intelligence and data mining [10],[23]–[25] and investigations that can be activated by signals from other sources such as patients, insured peoples, employees or health care providers (i.e. ‘whistle blowing’) [11].

#### c. Interventions aimed at responding to health care fraud

Response refers to administrative and legal actions based on the detection and investigation of the fraudulent cases in order to redress the lost money, fine the fraudsters, and sanction legal punishments to prevent future frauds. It may also involve changing and improving the system or law enforcement initiatives so that the chances of future frauds are reduced [11],[26],[27].

### Selection process, data extraction and analysis

Two authors (HJ, AR) first assessed the titles and then the abstracts to exclude the articles that clearly did not meet the inclusion criteria. We ordered the full texts of the remaining articles for assessment, and assessed the papers eligibility for inclusion. Disagreements were resolved via discussion and consensus. We assessed the quality of the included studies via assessing the following variables: allocation procedure (sequence and concealment), outcome measurement at baseline, similarity of characteristics at baseline, completeness of outcome data, preventing knowledge of allocated intervention, protecting against contamination, selective outcome reporting, and other risks of bias. One author (HJ) extracted data from the included studies, and another author (AR) assessed the completeness and accuracy of the data extraction. Given that few studies were found that used different methods and interventions, we used a narrative synthesis approaches for the interpretation of the studies [28] (See Appendix S2).

## Results

**Figure 1:**
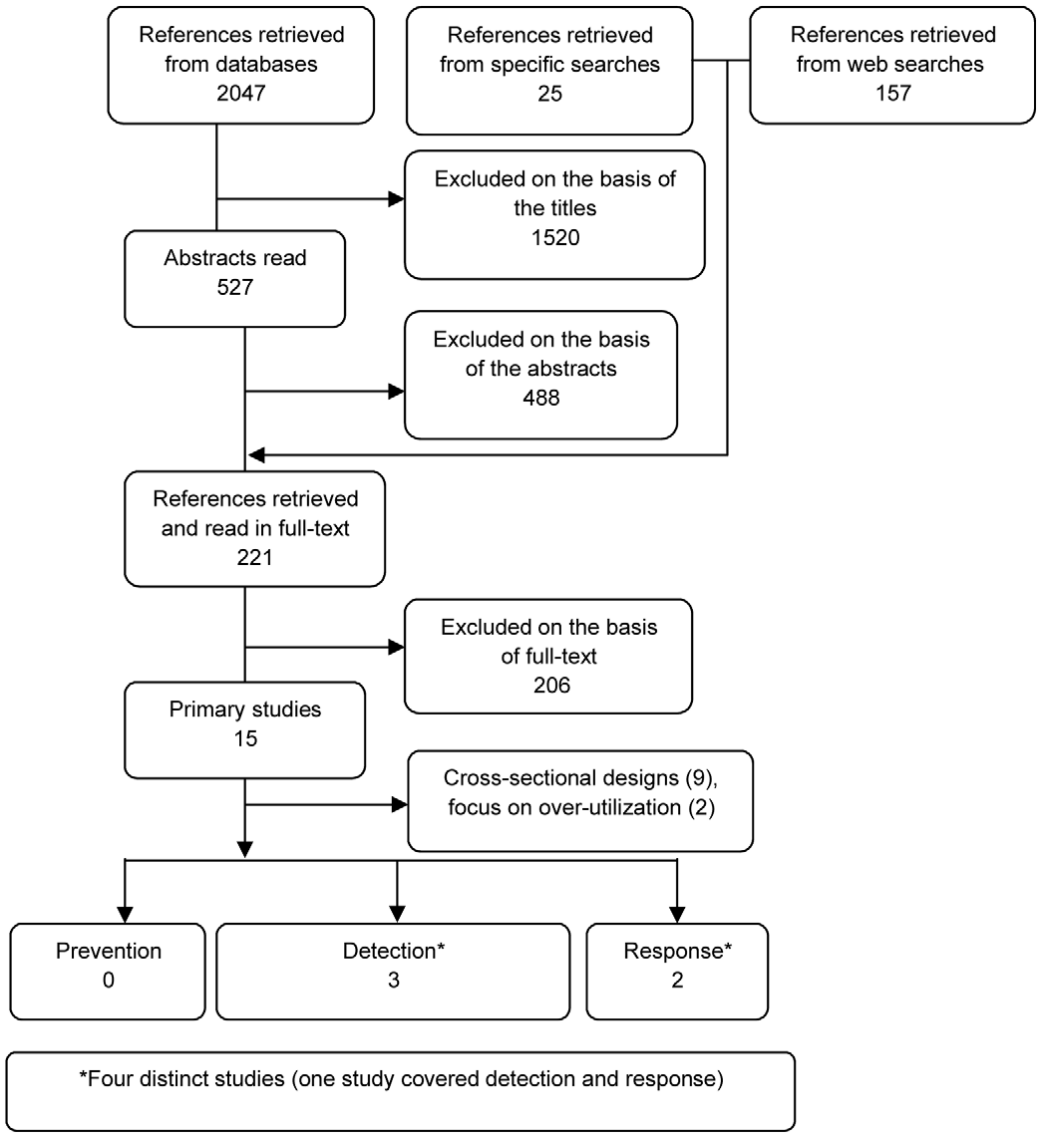
Paper selection flowchart.

We assessed 2229 titles and 527 abstracts. 221 full text reports and papers were examined (See Figure 1). We found four original articles that met the minimum inclusion criteria (See Table 2). We found no study under the prevention category. We identified two studies in the detection category [29],[30] and one in the response category [16], and one study that spanned both detection and response categories [31]. The included studies were very limited in their designs, and as such they were all subject to potential important biases. None of the studies incorporated a high quality interventional (e.g. RCT) or quasi-experimental (e.g. before-after controlled trial or interrupted time series) research design. Still we assessed the quality of the studies using a simple assessment tool (results available upon request). Based on the quality assessment, Becker et al 2005 study provides a more robust research method compared to the other studies [31].

**Table 2:** List of the included studies.

First author and date	Topic	Category	Journal	Country	Research design
Becker2005 [31]	Intensity of interventions to reduce abuse	Detection and response	Journal of Health Economics	USA	Longitudinal with concurrent control group
Liou2008 [29]	Detecting hospital fraud and claim abuse in diabetic outpatient services	Detection	Health Care Management Science	Taiwan	Data mining
Yang2006 [30]	detection of health care fraud and abuse	Detection	Expert System With Application	Taiwan	Data mining
Rivers2005 [16]	Effects of clinical laboratory improvement amendment of 1988	Response	Health Care Management Review	USA	before-after study

We first report the findings of the ‘evidence’ identified in our study for each category of anti-fraud interventions. As we identified very little evidence answering the effectiveness questions, for each category of interventions we provide examples of the findings of related (not included) studies to describe relevant aspects of the current thinking about the intervention. We clearly mark this latter group under the title ‘related studies’.

### Prevention

#### Evidence

We found no studies that assessed the effectiveness of interventions in preventing health care fraud and abuse.

#### Related studies

A few studies assessed the physicians' and public perception of fraud. These studies showed that in some situations at least a fraction of physicians and patients justified and accepted committing acts of deception. For example, studies in the USA showed that from 10% to about 40% of physicians reported manipulating reimbursement rules or their willingness to manipulate such rules to enable patients to receive the care that the physicians perceived as necessary [32]–[35]. More importantly, a large survey of prospective jurors and physicians in the USA showed that the public was more than twice as likely as physicians to allow deception that might benefit patients (26% versus 11%) [37],[38]. Further, it seems public support for deception (as compared to physicians) may have increased over time [36],[37].

### Detection

#### Evidence

This category refers to the interventions that result in improved detection and confirmation of the fraud. We found three studies with original data, two studies from Taiwan, and one from the USA. None of the studies compared the effects of using a detection method in a health system with a control group that did not benefit from such detection approaches.

The two studies from Taiwan used data mining methods for identifying health care fraud [29],[30]. One study applied and compared three data mining algorithms — logistic regression, neural networks, and classification trees— on the claims submitted to Taiwan's National Health Insurance to detect fraudulent or abusive utilization of diabetic outpatient services [29]. This study used expenditure-related variables for comparing fraudulent and non-fraudulent claims and designing the detection models. The study concluded that the three algorithms were accurate in detecting previously known fraudulent claims [29]. However, the methods used in this study had important limitations. First, it seemed that the same set of data had been used for initial tests of the models as well as the final applications of the models. Using the same set of data results in an overestimation of the ability of the models in detecting fraudulent cases. Second, they did not used any other investigation methods (apart from data mining) to assess the accuracy of identifying fraudulent cases other than relying on the previous verdict of the routine assessments conducted by the insurance system. Hence, their accuracy rates were only about whether the data mining approach corroborated the previous judgments of the insurance system.

Another study, also using Taiwan's National Health Insurance data, used data mining to see whether the providers followed the previously defined clinical pathways, and used deviations from clinical pathways as an indication of potential fraud or abuse [30]. They assumed that normal care should follow a logical sequence and be performed in the order defined by the clinical pathway. They provided a detailed description of how such logics were incorporated into a data mining structure for fraud detection. To test their model, they selected a relatively straightforward clinical scenario (pelvic inflammatory disease) and used the judgments of two specialists who assessed over 2500 claims and identified about 900 cases of ‘fraudulent’ behavior. The authors also randomly selected about 900 cases from the remaining ‘clean’ claims as the control group. Then the model was used to see whether it was capable of correctly identifying clean and fraudulent cases from each other. The best sensitivity and specificity rates of detection in their models were at 64 and 67%, respectively [30]. Hence, this study used expert judgments and investigation approaches, rather than relying on routine assessments, for identifying the potentially fraudulent and clean cases. Then they used the logics of the pathways of care for the identification of fraud using data mining. As a limitation, the authors' assumption that normal care should follow a logical sequence might not reflect the majority of clinical care, and hence the proposed approach may result in false alarms and detections. The proposed approach should be tested on new sets of data and see whether it can effectively enhance the ability of detecting fraud.

A third study estimated anti-fraud activities in different US states, and then linked that with fraudulent activities in the states [31]. This comparative study aimed to detect fraud and abuse in the Medicare and estimated the amount of anti-fraud expenditure at the state level per hospital and per patient as a measure of intensity of anti-fraud interventions in the state. The study covered issues relevant to the ‘detection’, and ‘response’ categories of interventions to combat health care fraud. We provide further detail about this study under the ‘response’ category.

#### Related studies

Several studies have described the importance of data mining and different steps that should be followed in using this approach for health care fraud and abuse detection [10]. Among these, one study conducted in the USA explains the steps that should be followed in any data mining approach, especially the introductory steps of preparing and visualizing the data [38],[39]. They provide detailed examples of data mining using Health Care Financing Administration claims related to preventative services of mammography, bone density assessment and diabetic counseling [38],[39].Their study demonstrates how such data cleaning and visual and descriptive analysis can identify suspicions related to abuse and fraud. They distinguish four data mining approaches: normative profiling (identifying the providers not following the usual patterns of care), abuse and misuse profiling (identifying joint groups of patients and providers not following patterns of appropriate behavior), change detection (focusing on important changes in providers' billing practices), and link analysis (focusing on referral and linkage patterns between different providers and their patients). Their approach is useful for fraud detection for individual providers as well as beneficiaries; however, the authors do not provide any data on accuracy rates in fraud detection or the advantages of their approaches over other detection methods [38],[39].

### Response

#### Evidence

We found two articles in this category, both from the US. The first study used a before-after study design to assess the effects of a legal intervention on fraud in diagnostic laboratories [16]. The main finding of this study was that more lenient sanctions from 1997 to 2001 in the USA correlate with the gradual increase in the percentage of fraudulent activities in the same period. This study, however, had no control groups and a high risk of bias.

Another study estimated anti-fraud activities in different US states, as mentioned above under the ‘detection’ category [31]. The researchers' main objective was to assess the association between anti-fraud interventions in US states and the observed amount of fraud and abuse relevant to certain disease groups and in different hospitals. The study focused on Medicare data and used the amount of anti-fraud expenditure at the state level per hospital and per patient as measures of the intensity of anti-fraud interventions. Their main conclusion was that more intense anti-fraud activity results in generally less occurrence of health care fraud and abuse. They observed that increased anti-fraud expenditure was linked to decline in billing by certain types of patients and hospitals without adverse consequences for patients' health outcomes.

A main limitation of this study is in the way that the interventions have been defined, i.e. by the amount of money spent on anti-fraud and abuse activities relevant to detection and response, without clearly specifying the interventions used [31]. It was also unclear whether the different states used the money on the same set of interventions or on different interventions. Hence although its findings are important and encouraging, it is difficult to make clear policy conclusions from its findings [31].

## Discussion

We conducted an extensive search and assessed several papers and reports on prevention, detection, and response to health care fraud. The results of the study yielded very few relevant studies. We found no studies with RCT, controlled before and after, or interrupted time series designs assessing the effectiveness of interventions to combat health care fraud. However we found four original articles that assessed different interventions using less robust research methodologies: two studies within the detection category, one in the response category, one under the detection and response categories, and no studies under the prevention category. As such the main finding of this research is that there is little published evidence on the effectiveness of interventions to combat health care fraud. Although the literature has mainly focused on provider fraud [10], we demonstrated that even for this group the literature is patchy and many questions remain unanswered.

Our searches were conducted on ‘health care’ databases, and we may have missed research published in criminal justice or law journals. Still our further searches in several relevant websites and reference list searches failed to identify relevant effectiveness studies. As such, it seems that the findings of our study represent the current state of effectiveness studies of interventions to combat health care fraud. Our analysis shows that there is a lack of research assessing the effectiveness of intervention to combating health care fraud, and the limited studies available are not using robust research methods. Although there are high profile reports and analyses focusing on the scale of the problem due to increasing national, regional and international fraud control initiatives [4],[40],[41], we are not any wiser regarding the effectiveness of interventions.

One reason for this gap is that the nature of fraud makes it difficult to conduct effectiveness studies in experimental or controlled settings [42]. Yet, examples of such studies exist in other related areas such as corruption in the provision of public services [43] and in insurance fraud [44]. In the latter, Blais and Bacher (2007) conducted a randomized trial to assess the effects of a letter to the insured persons that reminded them of the punishments for insurance fraud on fraudulent claims asking compensation for residential theft and concluded that the experiment was effective in reducing fraud [44].

Another possible reason for this observed lack of evidence is that enough attention has not been given to the health care fraud as an academic issue. Or perhaps the scale of health care fraud in the eyes of many policy makers and clinicians is not large enough to make it a priority issue among the long list of other priorities that health systems face.

Our review also revealed another important shortcoming in health care fraud research. We found no study from low income countries and very few studies from middle income countries, although such countries might be more prone and vulnerable to health care fraud and its consequences [7]. We also noted that the efforts to combat health care fraud are mainly focused on public expenses whether by government or by insurance organizations. Even less attention is paid to fraud and abuse related to private insurance organizations.

While we noted lack of evidence on the impact of fraud control interventions, we identified several studies that documented the problem. For example, a review of case studies conducted in 33 organizations from six countries concluded that the ‘percentage loss rate’ due to fraud and abuse in health care ranged between three to ten percent with an average of 5.6% of total health care costs [4]. All the studies cited in their review originated from high income countries [4]. As another example, a separate systematic review of the literature published in Farsi language [22] identified a limited number of primary studies focusing on fraud and abuse related issues in Iran that had not appeared in English language journals. That review, however, did not reveal any experimental studies. Our search was not restricted to any language, and we did not find any relevant study published in other languages. Still there may be relevant publications in other languages, as our search was limited to studies with at least a title or an abstract in English language.

Among the studies that focused on estimating health care fraud and abuse prevalence and rate, a report from the UK provides details of the methods used in their assessments. Even in this study, the losses due to fraud and abuse are mixed with losses due to abuse (referred to as ‘errors’ in the report) [42]. Hence further studies on assessing the scale of fraud and designing robust methods for such assessments are required. Previous studies have noted that many published estimates of fraud prevalence and loss may not be reliable because of methodological limitations [9].

There was a tendency of over reliance on computerized methods (e.g. data-mining) among the studies that focused on better detection of fraud. Excessive reliance to highly automated systems for fraud detection is not all of the solution. For example if a fraudster bills correctly for one or a group of patients that have not received any services (a well documented deception), probably most automated systems could not recognize it as fraud. We suggest that in detection systems, regardless of the extent of automation, a representative sample of patients (claims) should be tracked down and scrutinized as part of the fraud detection strategy. Such assessments might involve contacting providers or patients, if there is a reasonable likelihood of fraudulent behavior.

We found no study assessing interventions that focused on fraud prevention strategies. Participants in an expert opinion survey in the USA believed that heavy penalties are potentially the most cost-effective legal approach to combat health care fraud and abuse [45]. While no robust research evidence exists, criminology studies and deterrence theories provide useful lessons which may be applicable to health care fraud and abuse [44]. Such theoretical perspectives might be considered in designing interventions to combat fraud.

Understanding the nature and the scale of fraud is a pre-requisite in any attempts to combat health care fraud. Accurate estimates help prioritizing the interventions and justifying the amount of resources required for combating fraud. Further studies using robust research methodologies are required in all aspects of dealing with health care fraud, assessing the effectiveness and cost-effectiveness of methods to prevent, detect, and respond to fraud in health care.

## Supporting Information

Appendix S1 **Search strategy.**
(DOC)

Appendix S2 **PRISMA 2009 Checklist.**
(DOC)
