# Supplementary material for: No Evidence of the Effect of the Interventions to Combat Health Care Fraud and Abuse: A Systematic Review of Literature
Source: PLoS One. 2012 Aug 24;7(8):e41988. doi: 10.1371/journal.pone.0041988 (PMC3427314; doi:10.1371/journal.pone.0041988)
Supplement: Appendix S1 — Search strategy. (DOC) [file pone.0041988.s001.doc]

Appendix S1: search strategy

Database: Ovid MEDLINE(R) <1950 to January Week 2 2010>

Search Strategy:

--------------------------------------------------------------------------------

((Randomized controlled trial/ or (randomised or randomized).tw. or experiment$.tw. or (time adj series).tw. or (pre test or pretest or post test or posttest).tw. or impact.tw. or intervention?.tw. or chang$.tw. or evaluat$.tw. or effect?.tw. or compar$.tw.) not (animal/ not (animal/ and human/))) and (exp fraud/ or fraud$.mp. or exp Forgery/ or ((health or medical) adj2 (fraud$ or misconduct or decept$)).mp. or ((finance$ or bill$ or contract$ or claim$) adj2 (Falsif$ or abuse or misrepresent$ or unbundl$ or theft or Forgery or misconduct or crim$ or corrupt$ or misguide)).mp. or (Kick?back$ or kickback$).mp. or ((false or falsif$ or fraud$ or crim$) adj2 (claim$ or schem$)).mp. or (((excessive or over) adj2 utili?ation) or overutili?ation).mp. or ((Falsif$ or fraud$) and diagnos?s).mp. or Fee?splitting.mp. or Staged?accident.mp. or quackery.mp. or (rent$ adj patient$).mp. or counterfeit.mp. or bribe.mp. or upcoding.mp. or self?refer$.mp.) [mp=title, original title, abstract, name of substance word, subject heading word, unique identifier]
